# Hope and meaning-making in phase 1 oncology trials: a systematic review and thematic synthesis of qualitative evidence on patient-participant experiences

**DOI:** 10.1186/s13063-022-06306-9

**Published:** 2022-05-16

**Authors:** Kate Escritt, Mala Mann, Annmarie Nelson, Emily Harrop

**Affiliations:** 1 Cardiff University Division of Population Medicine, Neuadd Meirionnydd, Heath Park, Cardiff, UK; 2 SURE/Marie Curie Research Centre, Cardiff University Division of Population Medicine, Neuadd Meirionnydd, Heath Park, Cardiff, UK; 3 Marie Curie Research Centre, Cardiff University Division of Population Medicine, 8th floor, Neuadd Meirionnydd, Heath Park, Cardiff, UK

**Keywords:** Cancer, Palliative Care, Phase 1 trials, Qualitative, Coping, Consent

## Abstract

**Background:**

Phase 1 drug trials are popular treatment options for patients with advanced disease, despite the greater levels of uncertainty associated with them. However, their meaning and consequences for patient-participants remains under-explored. This review synthesises the qualitative evidence of patients’ experiences of participating in phase 1 oncology trials, exploring their decisions to take part and the impacts of these trials on patient wellbeing.

**Methods:**

A comprehensive literature search involving medical subject headings (MeSH) and keywords was undertaken in the following databases: MEDLINE, EMBASE, PsycINFO, Scopus, CINAHL, and Cochrane CENTRAL, with supplementary searches also conducted. Studies were independently screened for inclusion by two researchers. Included studies were critically appraised and data extracted using standardised forms. Qualitative results were analysed using thematic synthesis.

**Results:**

Three main themes were identified across 13 studies: decision-making and joining the trial; experiences of taking part in the trial and hope and coping. Patients primarily joined trials hoping for therapeutic benefits, sentiments which prevailed and shaped their experiences across their trial journey. Rather than indicate therapeutic misconception based on poor understanding, patient perspectives more commonly pointed to differences between hope and expectation and cultural narratives of staying positive, trying everything and trusting in experts.

**Conclusions:**

These findings challenge information-based models of consent, favouring coping frameworks which account for the role of hope and meaning-making during serious illness. Personalised consideration of existential and quality-of-life matters before and during trials is recommended, including palliative and supportive care alternatives to active treatment.

**Review Registration:**

The review was registered with PROSPERO international prospective register of systematic reviews (CRD 42020163250).

**Supplementary Information:**

The online version contains supplementary material available at 10.1186/s13063-022-06306-9.

## Background

For patients with advanced cancer, the decision to participate in a clinical trial is complex. Hope and meaning become especially important in patient efforts to cope with their illness and changing life situation [1–6], with treatment decisions featuring centrally within these narratives [2, 7–10]. For some, priorities may change to focus on quality rather than quantity of life [2, 7], whilst for others, survival is the ultimate goal and trials are seen to offer the best chance of achieving this [8].

Phase 1 (P1) drug trials are essential for improving cancer treatment; however, the experiences, costs and benefits to individual participants remain relatively unexplored [11]. Unlike later phase trials, these ‘first-in-human’ studies aim to establish the safety and maximum dosages of new medications rather than drug efficacy [11, 12]. Traditionally, P1 trials have not presented viable therapeutic benefits for participants [13] and patients frequently overestimate the anticipated effects of treatments [14]. However, due to advancements in cancer care and scientific understandings, there may be greater potential for therapeutic responses in current early-phase studies [15]. Despite these advancements, P1 trials remain contentious as treatments as they are far from proven and are not risk-free; exposure to untested agents presents risk of toxicity and unknown side effects. Modern monitoring has reduced toxicity [16], but serious adverse events, including fatal incidents, do still occur [17]. Further, these trials often have high take-up amongst patients with advanced-stage disease who have exhausted mainstream treatments [11, 12].

Ethical concerns relating to lack of informed consent in clinical trials have been identified. Patients have been shown to have misunderstandings about trial purpose and process [9, 18], difficulties accepting equipoise [19], therapeutic misconception and the overestimation of possible benefits, as well as limited recall of risks or disadvantages of trials [9, 20]. Unrealistic optimism is also a concern, whereby patients might understand the risks and benefits associated with the trial, but sustain a belief that they are more likely to benefit/less like to suffer harm than others in the same situation [21]. Trust in healthcare professionals and expectations of personalised care are also highlighted as factors influencing decisions to join or decline trials [9, 19, 22–25] and a corresponding need for more ‘relational’ conceptualisations of autonomy for understanding patient choices in healthcare contexts has been proposed [18]. The importance of person-centred recruitment approaches has also been identified in recent reviews of decision-making in healthcare trials [26, 27].

In the context of patients with advanced-stage disease, where treatment options are limited and trials are seen to offer hope by giving access to new treatments, it has been suggested that patients experience enhanced vulnerability [10, 28]. Dellson et al. observed how their palliative patients’ decisions to participate in trials seemed ‘instant’, guided more by emotion than deliberation, and were based on positive feelings towards their doctors and medical research in general [10]. A recent review of decision-making in cancer patients contemplating trials of any phase showed hope of therapeutic benefit as key to participation [12]. It demonstrated the central role of hope and existential considerations in patient decision-making, observing how clinical trials can equate with hope for patients, where, for many patients, treatment becomes the meaning in life, a way to try to live and a hope to the end [12]. The importance of hope is widely recognised in the cancer and palliative care literature [1–5], but this does not always equate with hope of recovery. Alternative hope and meaning-making is described for people approaching the end of life, when one’s life priorities and goals are re-evaluated, with associated calls for such considerations to feature more centrally in treatment and trial consultations at this time [2, 7, 8, 10]. These should include discussion of the benefits of specialist palliative care, which may sometimes be perceived as ‘at odds’ with trial participation and associated hopes for recovery [29, 30].

These observations suggest the value of meaning-based coping frameworks for the study of patient decision-making and trial/treatment experiences. Folkman’s Stress and Coping Theory demonstrates hope and coping as reciprocal factors, each supporting and supported by the other [31]. Folkman defines stress as a situation which is personally significant and exceeds the person’s capacity for coping, enforcing people to use different coping mechanisms, such as the meaning-coping mechanism [31]. Meaning-based coping is often seen in end-of-life cancer patients whereby patients’ reorder their priorities based on deep-rooted values [31]. Stress and Coping Theory shows hope as essential for those with prolonged stressors as hope sustains long-term coping [31]. Another framework applied to cancer patient coping is Antonovosky’s Sense of Coherence (SoC) theory [2, 4, 6, 32–34]. This theory describes how people maintain wellbeing through times of adversity and is made of three factors: comprehensibility, manageability and meaningfulness [35]. Comprehensibility refers to how people see the world and their ability to understand what happens around them. Meaningfulness refers to the way in which the person finds meaning in the situation and sees the demands or stressors as challenges worthy of emotional investment, whilst manageability describes the extent to which they can respond to the situation and perceive resources available to enable them to respond [32]. When studying the treatment decisions of patients with advance disease, this framework has the advantage of enabling due consideration of the cultural narratives and core life concerns shaping such decisions. As such it also supports more contextualised approaches to decision-making and consent processes, which have been shown to be needed in place of overly information-based or cognitive models [9, 18].

Whilst recent reviews have considered the evidence relating to patient decisions to join oncology trials [8], none have considered participant experiences of the whole P1 trial process, including impacts on patient coping and wellbeing. This is especially important given the potentially larger risks associated with these trials and the fact that quantitative measures of patient experience, such as quality of life, are not typically used. This review aimed to synthesise the qualitative evidence regarding cancer patient experiences during P1 trials from recruitment to post-trial follow-up, encompassing the entire participant experience.

## Methods

The objectives of this systematic review are to explore participants’: reported motivations for enrolling in P1 trials, understandings of trial purpose and process, perceived risks and benefits, side effects and quality of life and overall experiences. This systematic review was registered with PROSPERO international prospective register of systematic reviews (CRD 42020163250) and reported in accordance with PRISMA guidelines [36].

### Search strategy

A comprehensive literature search employing both MeSH headings and keywords was undertaken in the following databases: MEDLINE, EMBASE, PsycINFO, Scopus, CINAHL and Cochrane CENTRAL, from database inception to December 2019, and updated in February 2021 (Supplementary file one: search strategy). The topic lent itself to three sets of search components: phase 1 trials, cancer and patient experience. The Information Specialists’ Sub-Group (ISSG) Search Filter for Qualitative Research was applied as part of the ‘experience’ set [37]. We also searched BioMed Central, and International Randomised Controlled Trial (ISRCTN) registry and unpicked eight relevant systematic reviews for further primary studies. Reference list checking, citation tracking and contacting authors of included papers were conducted.

### Study selection

Identified papers were imported into EndNote for duplicate removal before titles and abstracts were independently screened against predefined inclusion criteria by two authors (Table 1). Screening results were compared and agreements reached on which studies to consider further. Full texts of the selected studies were further screened by both researchers. Thirteen studies (14 papers) were considered eligible for use in the review.

**Table 1:** Inclusion criteria

	Inclusion	Exclusion
Population	Adults who consent to take part in a phase 1 trial (trial participants) Oncology patients Any type of cancer/ stage of disease Any location in world (due to lack of research in the area)	Children (under 18 years) Healthy volunteers
Interventions	Phase 1 cancer drug trials	Phase 1 trials of non-drug interventions Drug trials not in phase 1 Phase 1 trials for non-cancerous diseases
Outcomes	Patient views and experiences Qualitative data	Quantitative data

### Critical appraisal, data extraction and synthesis

Two reviewers assessed study quality using the SURE qualitative checklist [38]. Using the criteria in the checklist, agreement was reached on which studies should be considered ‘good’, ‘mixed’ or ‘low’ quality. Data were extracted using standardised, pre-piloted forms (Supplementary File 2). This enabled summarisation of each study characteristics and results and informed the categories in the included studies table. One reviewer extracted the data and the second reviewer completed data extraction on 20% of papers to ensure quality and validity.

Included papers were uploaded to NVivo for analysis and data management. Thematic synthesis was undertaken by the lead researcher (KE) coding each line of relevant results (the qualitative themes described by study authors) for meaning to allow study concepts to be translated to other studies [39]. Participant extracts reported in study papers were also coded according to the themes which they exemplified. Through the analysis process, individual lines of the papers were coded and grouped together with other similar codes, before identifying common labels (descriptive themes), which were agreed upon through discussion with co-author EH. Once descriptive themes were established deeper data inferences could be drawn, allowing analytical themes to become apparent. Final themes were reviewed by co-authors, ensuring review integrity and validity.

## Results

### Study characteristics and methodological quality

**Fig. 1:**
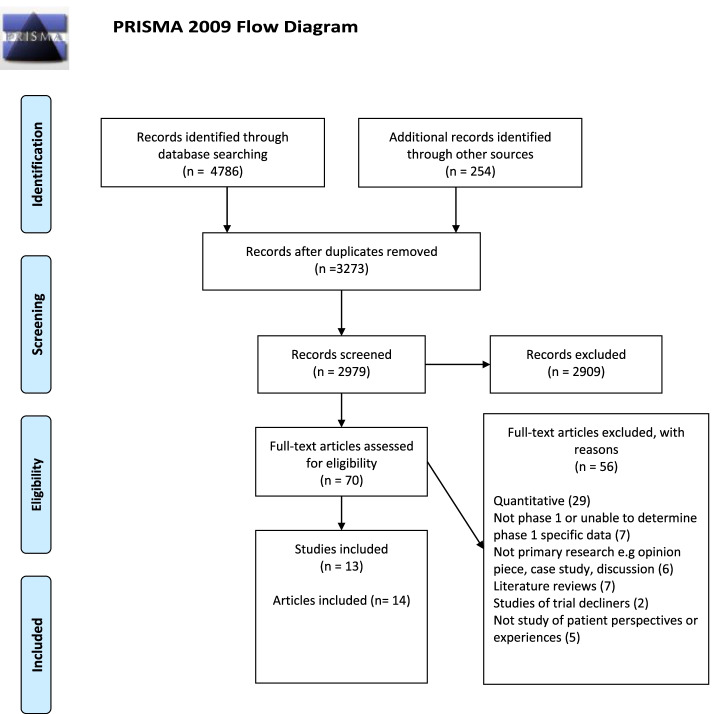
Screening results

Fourteen papers, from thirteen studies, were included [40–53] (Fig. 1: Flow diagram). These studies used interviews, focus groups and mixed methods surveys with a combined total of 328 participants covering a range of cancers (Table 2: Included studies). Seven studies fulfilled almost all quality criteria and could be considered good quality [40, 41, 43–46, 50, 53], the other studies were either of mixed [47–49, 51, 52] or low quality [42]. The reasons studies were considered lower quality were as follows: lack of rigour demonstrated in analysis [42, 48, 51, 52], very small sample size [47] and limitations and lack of detail concerning data collection [42, 49, 51, 52]. Where observations from the one low-quality study are reported in isolation, the poor study quality is made explicit in the narrative.

**Table 2:** Included Study Characteristics

Study	Aim	Design	Population	Data collection	Key findings
Bredart, 2017 (France) [40], Good quality	Describe perceived side-effect tolerance in P1 trials.	Qualitative	17 patients 12 female, 5 male Aged 41–72 years (median 63) Cancer type: melanoma, breast, nasopharyngeal, cervical, endometrial	Face-to-face semi-structured interviews of open questions.	As trial is last treatment hope, patients accept side effects, resulting in reduced reporting. Patients stop trial treatment if it stops working rather than side effects. Disappointed when it is not effective.
Cohen, 2007 (USA) [41], Good quality	Describe the burdens and benefits, as well as perceived QoL, of P1 trial patients.	Mixed methods: Survey with some patients interviewed	16 patients 10 male, 6 female 29–69 years (57 mean) Cancer type: solid tumours (not specified)	Face-to-face interviews audiotaped and transcribed.	Patients’ QoL was good as they were free from cancer symptoms or drug side effects. However, the trial process was a huge burden as they were away from home and had to spend a lot of time at the hospital for treatment.
Daugherty, 1995 (USA) [42], Low quality	Understand patient perceptions of P1 trials, and issues related to their participation.	Mixed methods: Survey with both open and closed data	27 patients 19 male, 8 female Aged 32–80 (median 58 years) 70% white; 26% African American Cancer type: 15 different diagnoses (not specified)	Structured interviews of open and closed questions. Responses hand-written.	P1 trial participants are strongly motivated by hope of therapeutic benefit and very few patients understand the purpose of P1 as dose-determination studies.
Ferrell, 2019 (USA) [43], Ferrell, 2020 (USA) [44] Good quality	Capture patient perspectives of P1 trial participation and disease/ treatment options [43]. Secondary analysis focused on spiritual needs of this population [44].	Qualitative	30 patients 56.8% female 30.7% ethnic minority Aged: <40 = 3, 50–59 = 8, 60–69 = 9, 70–79 = 8, >80 = 2 Cancer type: lung, bladder, colon, ovarian, prostate, breast, cervical, other	Interviews audio-recorded and transcribed.	Doctors, lack of other options, altruism and family motivate patients to join P1 trial. Patients’ expectations of trial are to get better, improve their QoL, and reach remission or cure. These motivations are optimistic not misconceptions [43]. The transition to phase 1 trial participation is a time of balancing hope for extended life with the reality of disease [44].
Godskesen. 2013 (Sweden) [45], Good quality	Explore patients’ reasons for participation in, and experiences of, P1 trial participation.	Qualitative	14 patients Male 9, female 5 Age: range 51–81 (median 63) Cancer type; prostate, melanoma, lung, pancreas	Face-to-face semi-structured interviews audio-recorded and transcribed.	Patients had poor understandings of the trial and demonstrated therapeutic misconception. Hope of trial success was good for patient wellbeing and mental health. Trial offers patients extra care and attention which was a positive factor.
Kohara, 2010 (Japan) [46], Good quality	Understand the decision-making process in participation of P1 trials	Qualitative	25 patients Male 14, female 11 Age: <50 = 5, 50–59 = 7, 60–69 = 10, >70 = 3 Cancer type; colon, lung, breast, head and neck, renal, oesophageal, pancreas, biliary tract, ovary, liposarcoma, thymoma	Face-to-face semi-structured interviews audio-recorded and transcribed.	Decision-making depends on: doctors’ influence, previous experiences, attitude towards cancer, family (biggest influence)
Kvale, 2010 (USA) [47], Mixed quality	Appreciate the experiences of older adults in P1 trials	Qualitative	4 patients Male 3, female 1 Older adults—mean age 63 Cancer type; lung, lymphoma, paraganglioma	Face-to-face semi-structured interviews audio-recorded and transcribed.	Patients use social comparison and hope to aid them through the process
Moore, 2000 (UK) [48], Mixed quality	Capture patient perceptions of P1 participation	Qualitative	15 patients 12 female, 3 male Cancer type; 9 different diagnoses (not specified)	Open-questionnaires and an interview audiotaped and transcribed.	Patients felt a need to try everything at any cost. Patients understood the reality of the disease while hoping to be cured. Trial benefits participants and future patients
Pentz, 2012 (USA) [49], Mixed quality	Determine if patients misunderstand trial info and identify those who suffer therapeutic misconception	Mixed methods: Interviews followed by a survey	95 patients 53 male, 42 female median age 57 (range 28–85) 67% white Cancer type: not specified	Interviews audio-record and transcribed.	Therapeutic misconception associated with lower income and higher education. Most participated with hope of direct medical benefit, although other motivations also included: altruism, doctor’s recommendation, other collateral benefits of trial.
Reeder-Hayes, 2017 (USA) [50], Good quality	Understand patient decision- making to enter trial	Qualitative	18 patients Female Cancer type: metastatic breast cancer	Telephone semi-structured interviews audio-recorded and transcribed.	Family is a powerful motivating factor, patients join trials for therapeutic gains as well as other factors.
Rodenhuis,1984 (Netherland) [51] Mixed quality	Explore motives to partake or refuse P1 trial and evaluate quality of consent	Qualitative	10 patients 6 males, 4 female Cancer type: melanoma, head and neck, lung, breast, cervix	Face-to-face interviews.	Many patients did not understand the trial purpose but were motivated by disease improvement and their families.
Schutta, 2000 (USA) [52], Good quality.	Explore factors which influence the decision to join a P1 trial	Qualitative	8 patients Female 5, male 3 Range = 42–72 (years) Cancer type: lung, renal, breast, gastrointestinal	2 focus groups. 1st recorded (*n* = 6) and 2nd (*n* = 2) took notes.	Patients understand the trial purpose but choose to focus on hope of medical benefit.
Sulmasy, 2010 (USA) [53], Good quality.	Explore justifications for estimations of expected therapeutic benefit from p1 trials	Mixed methods	45 patients 23 female, 22 male Mean age 57 Cancer type: not specified	Face-to-face interviews audio-recorded and transcribed.	High hopes of therapeutic benefit had little to do with knowledge and more to do with expressions of optimism.

### Thematic synthesis

**Fig. 2:**
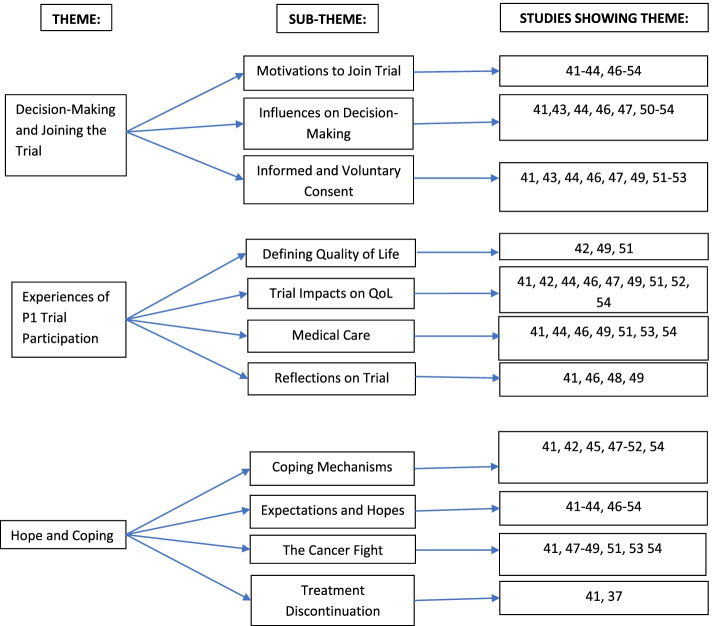
Theme diagram

Thematic synthesis identified three main themes and eleven sub-themes: decision-making and joining the trial, experiences of trial participation and hope and coping. These themes are depicted in Fig. 2: Theme diagram.

### Decision-making and joining the trial

#### Patients join trials hoping to benefit themselves and others

All studies reported hope of therapeutic benefit as the primary motivating factor [40–43, 45–53]. Patients hoped for a cure, life extension and better life quality without symptoms or side effects. Trials were frequently considered a last chance at treatment, so participants were willing to take risks [45, 46, 50, 52].

“I am willing to take that chance for the benefit.” (Participant) [50]

“There was virtually no drawbacks involved and, therefore, I decided to participate” (Participant) [45]

Another motivating factor was altruism [42, 43, 47–49, 52, 53]; participants recognised benefits to future patients and scientific advance. However, these selfless considerations were consistently stated as secondary motivations [42, 45, 48, 50, 53], giving insight to the outlook of trial participants with advanced disease whose underlying concern with survival is greater than altruistic ideals.

“Secondary I will be able to help others, but first and foremost, it is for my own sake.” (Participant) [45]

“If it doesn’t help me, maybe it’ll help the next” (Participant) [53]

In joining trials, patients must weigh-up motivating factors with perceived risks. Acknowledged risks included side effects, lowered life quality and uncertainty of novel medications [46, 49, 50]. Some patients did not consider there to be participation risks [45, 49].

“There was virtually no drawbacks involved and, therefore, I decided to participate” (Participant) [45]

#### Clinicians and family influence decisions to join trials

Significant others influence patients’ decisions. Studies discussed the essential role of clinicians in facilitating trial participation [42, 43, 45, 46, 49–53]. Doctors are pivotal in their patients’ decision-making due to deep-rooted trust [40, 43, 50, 52, 53]. It is believed clinicians have great insight into their patients’ specific situation and would not suggest something potentially harmful [53].

“[The physician] knows my body now and he knows what will work … ” (Participant) [53]

“Trust the doctors” (Participant) [40]

“The doctors would not advise this treatment if it were not effective” (Participant) [51]

Patients’ families influence decision-making. Patients can join the trial for their family in hope of gaining additional life, in particular parents of young children [50, 53]. Families encouraging and occasionally pressurising participation can also prompt patients to join trials [42, 43, 45, 46, 50, 51] particularly if they are unprepared for the patient’s death [51].

“My husband recommends that it is better to do everything I can.” (Participant) [46]

“I must take them … I must for my children” (Participant) [40]

#### The generally informed and voluntary nature of consent

Information provision regarding trial purpose, risks and benefits is generally good, ensuring many patients are well-informed [40, 42, 45, 46, 50–52]; however, participants do not always read or fully understand this information [45, 50, 51]. Information of alternatives to trial participation is often provided including other trials, healthcare abroad, and complementary medicines [40, 42, 43, 45–48, 50, 52]. However, in one low-quality study, it was observed that very few patients recognised no treatment or palliative care as an option [42].

“I was able to read through that (consent form) and kind of discern, you know, what the study was about, what the drug did” (Participant) [50]

Patients felt consent was voluntary and valued making their own decisions [42, 43, 45, 48, 52]. Clinician support is appreciated in decision-making, without participants feeling pressured by medical staff [52]. Some patients reported familial pressures [43], but the most frequent concern was perceived lack of options affecting voluntary consent [45, 48]. Patients knew they could decline the trial, but lacking alternatives meant they felt they had no choice.

“I think it’s the only choice I’ve got really” (Participant) [48]

“They (the Doctors) leave the decision to the patient.” (Participant) [52]

### Experiences of phase 1 trial participation

#### Defining quality of life when living with advanced disease

Patients with advanced disease value a quality of life normally taken for granted; baseline function is perceived as good quality [41, 48, 50]. Patients value not being bedbound or hospitalised, being with family and living free from psychological burden [41]. Participants accepted lower quality of life in the terminal stages of disease, accepting baseline functionality may be unattainable [41, 48, 50]. However, patients highly value some independence, including being able to make informed choices about their treatment options [41, 48, 50].

“I accept very little. But I want this very little. I don’t want Everest; [I’m] not a mountaineer. ” (Participant) [41]

“We’re quite willing to put up with that if it helps” (Participant) [48]

#### Variable side effects and procedural burdens impact quality of life

P1 trials significantly impact participants’ life quality. Side effects are pivotal in patients’ overall trial perceptions. Therapies with small side-effect profiles, compared to standard chemotherapy, help patients feel better [40, 41, 43, 45, 46]. The most recent study produced therapeutic gains enabling participants to be free from cancer symptoms [43]. However, some patients find a lack of side effects unsettling as physical responses are strongly associated with treatment efficacy [40, 51]. Some trials demonstrated the unpredictable nature of P1 treatments with a range in side-effect severity [40, 43], whilst in other trials all participants experienced strong effects [51]. Side effects are detrimental to quality of life, causing anxiety, social isolation and changes in self-image due to functional decline [40]. Despite the presence of side effects, many patients continue with trials. The difference between tolerable and intolerable side effects are frequency, possibility of symptom control, psychological impacts and hope of improvement [40].

“My friends comment on it that I’m looking better. Certainly I feel better.” (Participant) [41]

“Phase 1 trial got rather nasty, health wise, the medication. The side effects were pretty terrible” (Participant) [43]

Procedural aspects affect trial experiences. Participating patients can gain a sense of purpose through hope of personal and altruistic benefits and structure to their lives in the chaos of terminal illness [41, 48, 53]. However, others felt extremely burdened by time spent in hospital [41, 43, 50]. Some trials require patients to relocate nearer to trial centres at significant emotional and financial cost [41]. Overall, some medical contact and structure is welcomed, but frequent appointments, extended hospital admissions and relocation is detrimental.

“I thought it was brilliant … Everybody knows you and it's like home from home.” (Participant) [48]

“I am away from home and that is very, very difficult ... I have a high school daughter at home … ....” (Participant) [41]

#### Receiving good medical care and having faith in medical staff

Patients gave positive appraisals of medical care received during the trials [40, 43, 45, 48, 50, 53]. Additional attention and testing made patients feel safer than standard care [45, 50]. Placing trust in doctors allows participants to share their disease burden, easing stress and anxiety [40, 43, 50, 52, 53]. Faith in medical staff was expressed more frequently than religious faith, reflecting (‘Western’) societal shifts from spiritual to scientific trust [53].

“The nursing staff go over and above taking care of my physical needs. They are interested in me” (Participant) [43]

“I know you are keeping a good eye on me and maybe that will help” (Participant) [48]

#### Retrospective reflections on trial participation varied by individual outlook

On retrospective reflection, some participants were happy they joined the trial and would join another [45, 46, 48]; living by the philosophy of ‘nothing ventured, nothing gained’ [48]. Other participants were glad they joined this trial but would not join another [48]. Some patients regretted joining trials, feeling valuable time was lost [40, 48]. However, regret was not necessarily associated with strong side-effect profiles or other negative factors. Rather, differences in retrospective appraisal may be based on personal philosophy and outlook.

“I regret that we did not stop the treatment earlier because it was not effective ” (Participant) [40]

“Unless you try something, you’re not going to know” (Participant) [48]

### Hope and coping

#### Coping mechanisms and strategies

Patients adopt a range of approaches to cope with terminal disease and demands of the trial. Coping mechanisms fall into three categories: giving up control to fate / god or physician, making comparisons to other treatments or people, and hope. Two studies highlighted fatalistic outlook, whereby participants resign themselves to fate or god, reducing anxiety by accepting the situation is beyond their control [44, 50]. Other patients give trusted physicians control, releasing them from difficult decision-making, shifting some burden [40, 51].

“I don’t worry about that because there’s absolutely nothing I can do about what’s going to happen” (Participant) [50]

“I accept the faith. I accept that, you know, when it's time to go, it's time to go. And that's what it is” (Participant) [44]

Patients make comparisons to others and previous treatments. Social comparisons give patients strength as others’ situations are even worse than theirs [40, 44, 46, 47]. Participants believe their characteristics mean they will do better in the trial than others [46, 49, 53]. Patients are comforted by feeling better than during previous treatments [40, 41, 46]. Unpleasant side effects are often comparatively better than those endured through previous chemotherapy. Experience of worse situations enables patients to mentally minimise the side effects and cope better [48, 50].

“There’s always somebody that’s got it worse, there’s always somebody worse off. I’ve still got options to turn to” (Participant) [47]

“ (past experience of) Chemotherapy is like taking rat poison … it’s worse than having the cancer.” (Participant) [41]

#### Distinguishing hope and expectation in patient coping and commitments to trials

The included studies demonstrate participants’ reliance on hope for emotional wellbeing [40–43, 45–53]. Hope is a key motivator to join and then endure trials; retaining hope is essential. Patients hope for therapeutic benefits from the trial: cure, remission, life extension, tumour reduction, improved symptoms and functioning. Studies specifically distinguish between participant hopes and expectations of trials [40, 43, 46, 48, 50, 53]. Studies reached different conclusions about how well-informed patients were before entering the trials. Some studies showed good patient understandings [50, 53], or different levels of understanding for different aspects of the trial [42], whilst one study suggested poor patient comprehension [45]. Generally, patients accepted that trials were not expected to treat them; nonetheless, they hoped this would happen. Hope can co-exist with full awareness of the realities of terminal disease. Patients are not delusional or misinformed but choose optimism, holding onto hope of benefit. This mismatch between expectation and hope can be linked to a motivating factor of trial participation ‘taking a gamble’; patients do not expect to win the lottery but hope they have the winning ticket [45, 46, 50, 52].

“But the fact that there was hope, we grabbed it with both hands.” (Participant) [48]

“The trial’s purpose is not my purpose ” (Participant) [50]

#### The narrative of cancer as a fight

Throughout the included studies, a discourse exists regarding cancer and its treatment as a ‘battle’ [40, 47, 48, 50, 52, 53]. This narrative places expectations on cancer patients by society, family, clinicians and themselves. Patients are at ‘war’ with cancer and therefore must be soldier-like: strong, stoical, courageous and dutiful. It is patients’ duty to remain hopeful and positive despite their terminal diagnosis, meaning patients do not wish to complain about side effects or seem ungrateful for the trial opportunity [40, 46–48, 52]. Participants can feel obliged to join trials to actively fight the disease. These expectations correlate with the common belief that positive outlook will affect physical treatment outcomes [47, 53].

“it’s a battle, it’s my battle in fact: giving myself the courage to go further.” (Participant) [40]

“I did two tours in Vietnam and I was a cop for 27 years. They didn’t get me, so I’m not going to let this get me either” (Participant) [53]

#### Deciding when to terminate treatment

The decision to discontinue trials is not taken lightly due to the expectations placed on patients [40, 48]. Participants endure unacceptable side effects due to desperation to remain on the trial; therefore, discontinuation is often due to treatment failure rather than side-effect profile [40]. The decision to discontinue is frequently left to clinicians as patients are afraid to make a decision they could regret [40]. Patients are more accepting of discontinuation when instructed by authority than feeling they ‘gave up’ and dropped out [40]. Discontinuation of P1 treatments prompts feelings of disappointment, guilt, fear and relief [40, 48].

“I was relieved when treatment discontinuation was decided on but I was disappointed” (Participant) [40]

“So, it is better for me to go where I want to go before it is too late.” (Participant) [48]

“I wanted to go on to the end so that I wouldn’t have any regrets, I wouldn’t blame myself, I wouldn’t tell myself “I didn’t have enough courage” … in that way I wouldn’t feel guilty…” (Participant) [40]

## Discussion

This is the first systematic review to consider participant experiences throughout phase 1 oncology trials. Thematic synthesis of 13 studies identified reasons for joining and continuing with early-phase trials, as well as impacts on physical and psychological wellbeing. These reasons included hope of benefit, altruism, good medical care and the influences of clinicians, family and cultural discourse, whilst impacts on quality of life and retrospective reflections varied. Dominant across the patient trial journey was a concern with maintaining hope as participants negotiated the physical, psycho-social and existential challenges associated with cancer diagnoses. Due to their explanatory ‘fit’ with many of these themes, we use meaning-based coping and Sense of Coherence (SoC) theory as a framework for interpreting these findings, and for theorising and contextualising patient decision-making and participation in early-phase trials. Implications are identified for improving consent processes and trial consultations.

Across the three phases of the trial (enrolment, continuation and termination), there is consistency in the coping mechanisms and decisions taken by patients, which reflect the three components in SoC theory: comprehensibility, manageability and meaningfulness [32]. As in previous reviews, this synthesis shows hope of therapeutic benefits as a primary motivator to trial participation and continuation, with altruism a secondary concern [8, 27]. This apparent bias towards treatment benefit presents an ethical challenge to models of informed consent in phase 1 trials [21], as in later phase trials [8–10, 23]. However, rather than view this as an informational or cognitive problem, these findings point to the need for a more contextualised conceptualisation of patient understanding, which in line with Antonovsky’s concept of comprehensibility may be considered optimal when a person understands as much as they want to about a particular situation [2, 6, 32]. Whilst there was evidence of limited understandings amongst some patients, this synthesis also demonstrated reasonable information giving and good levels of understanding relating to the risks and benefits associated with participation. However, it also suggests that a patient’s need to understand the scientific ‘facts’ exists alongside a more powerful need for hope, which is essential to their coping [54]. Although in a palliative context hope and coping can be achieved in relation to quality of life [7, 31, 54], for many participants it was hope of cure or significant improvement that gave meaning to their treatment decisions [2, 8], whilst also enabling feelings of agency, control and a sense of manageability [2]. These findings also demonstrated that whilst participants hope for therapeutic benefits they do not necessarily expect this to be the case, reflecting Leung’s conceptual model of hope and expectation as two different, inter-linked constructs [55]. These patients are not delusional or misinformed but choose therapeutic optimism [56], which would appear different from ‘unrealistic optimism’ [21] in that although patients may hold onto hope of benefit, they also recognise that the ‘odds’ are stacked against them.

However, it is important to locate this type of hope and optimism within broader cultural narratives of staying positive and ‘trying everything’ [2, 9], seen also in the observed ‘battle talk’ of participants and associated societal expectations of cancer patients to ‘fight’ the disease. Although recent research has reported prioritisation of quality over quantity of life [57], the burden placed on patients by societal oncology-military rhetoric is also well documented [58, 59]. War analogy mandates patient engagement so has implications for patients and their care decisions [60]. Relationships and narratives of trust in healthcare staff were similarly shown to influence and give meaning to the treatment decisions of patients, as in studies of later phase trials [9, 19, 24, 25]. By acting in line with the perceived opinions or preferences of their health professionals, enrolment decisions become more meaningful if they can be viewed as fulfilling cultural and personal expectations of expert informed care [9, 18, 19]. Manageability can also be seen to be enhanced by these decisions, which are perceived to give greater control and responsibility to the patient’s physician, thus sharing their disease burden.

As trials progress, two fundamental concerns prevail: maintaining hope and quality of life. McCaffrey’s systematic review found palliative patients describe quality of life as being able to complete usual activities [54]. This review also suggests good quality of life for these patients as fundamentally concerned with maintaining function and relationships. As in previous studies, adapted expectations and prioritisation of life activities and commitments, such as being at home and spending time with family, help patients to establish a normality which is both manageable and meaningful [2, 4–7, 34]. Trial participants also find meaning and a sense of purpose through hope of personal and altruistic benefits, as well as added structure to their lives [2, 9]. Positive appraisal of symptoms or side-effect burdens relative to previous experiences or the experiences of others, and a perceived higher standard of medical care, supports comprehensibility providing further rationale for ongoing trial participation [2, 9, 54]. However, trial participation can also undermine quality of life, through heavy side-effect burden or frequent appointments and periods away from home and family. The importance of maintaining social and family commitments towards the end of life is well documented [2, 6, 7], but the procedural aspects of trial and treatment schedules appear often over-looked by patients before commencing [7].

Despite the presence of side effects and disruption to quality of life many patients continue with trials, demonstrating the dominance of hope for improvement above other concerns, and societal and familial pressures associated with not giving up. Whilst participation risks are considered in decision-making, participants do not focus on these once they have joined the trial, instead trusting in their doctors to make decisions relating to tolerability and treatment continuation. Discontinuation is more commonly due to treatment failure rather than side-effect profile and is easier for patients to accept when instructed to do so by their physician; patients do not want to feel regret or guilt for not continuing, but for some there is also relief when these decisions are taken. Retrospective reflections, however, are not related to side effects or procedure, but rather dictated by individual outlook; some draw on cultural discourses of ‘nothing ventured, nothing gained’ and are happy to have tried whilst others regret wasting time with the trial, perceiving a cost to their quality of life.

### Implications for policy and practice

Patients join phase 1 trials primarily out of hope for therapeutic benefit, despite often showing good levels of understanding of the uncertain or unfavourable risk/benefit ratios. As observed previously, this challenges information-based models of consent [21]. Likewise, the influence of trust relationships and expectations towards health care professionals, alongside powerful narratives of maintaining hope and trying everything, reiterate the need for more contextualised and relational models of risk and decision-making [9, 18, 19, 61]. It is important for health care professionals to give personalised consideration to value-oriented and ‘quality of life’-related questions when discussing trial information and treatment options [7, 8, 18, 26, 27], which may support decisions more closely aligned with patients’ everyday goals and priorities [7].

It is important to recognise expectation and hope as separate constructs. If clinicians have ensured good patient understanding, including discussion around quality of life and alternative options, they should not be unduly concerned if a patient voices hope of therapeutic gain. Hope is essential whilst facing terminal illness and medical teams must maintain a careful balance between realism and hope. However, this also means recognising that hope and meaning are not only derived from the prospect of recovery; palliative care and existential discussion can also support an alternative quality of life-related hope for patients approaching the end of life [2, 7, 8, 10, 62, 63]. Healthcare professionals must be aware of the influence they have on decision-making; discussion of supportive and palliative care can help mitigate the ‘try anything’ approach and perceived bias towards active treatment [10, 18]. Palliative care should therefore be made available to all patients with advanced-stage disease, regardless of whether or not they join treatment trials [29].

This review has shown that side effects can vastly alter experiences, whilst excessive time away from home can be a source of anguish. Procedural burdens must be fully discussed and minimised to enable patients to spend maximum time at home. Ensuring that safeguarding processes are in place from trial entry could also help mitigate adverse experiences and preserve quality of life. These should go beyond biomedical assessments to give consideration to patient wellbeing, with ongoing discussion of alternatives to the trial so that discontinuation continues to be seen as a credible and legitimate option.

### Strengths, limitations and implications for future research

The methodology of this review was rigorous, reliable and comprehensive. Through thematic synthesis of qualitative findings, detailed insights are provided into the lived experiences of phase 1 cancer trial participants. A limitation of this review is the lack of specified time-period for included studies, and the mixed quality of some included studies. Scientific advances and the rapidly evolving field of phase 1 cancer trials mean the trialled treatments are significantly different in recent studies than those undertaken previously (and will continue to change again), especially regarding therapeutic benefits, although consistent themes were identified across the wide timespan. It was also not possible to conclude any meaningful differences between types of cancer. The cancer types and their treatments included in the studies varied between and within studies, making it difficult to determine cancer or treatment specific themes (although again the commonality of experience is of note). This review also only considered patients who joined trials, the majority of whom (in the studies which reported this) were from white ethno-cultural backgrounds, with all except one study from the USA or Europe. It is important to explore the experiences of patients who decline P1 trial entry, as well as those from diverse ethnic and cultural backgrounds who are underrepresented in early-phase trials [27, 64]. This will enable a more complete understanding of the decision-making process and possible differences in the values and beliefs of those who accept and decline early-phase trials. Further research could also develop tools which support ongoing assessment of patient quality of life and wellbeing. Given that such a tool could serve decision-making as well as data collection purposes, and the relatively small numbers involved in phase 1 trials, conversational qualitative or mixed methods tools warrant particular consideration here.

## Conclusion

This review has identified the reasons for participants joining and continuing with early-phase trials, as well as impacts on physical and psychological wellbeing. Patients primarily joined trials hoping for therapeutic benefits, sentiments which prevailed and shaped their experiences across the whole trial journey. Rather than indicate therapeutic misconception based on poor understanding, patient perspectives more commonly point to conceptual differences between hope and expectation. Meaning-based coping and SoC theory helps us to understand patient decisions and commitments in the context of their need for hope, as well as wider cultural narratives that incline patients towards fighting disease and trusting in experts. Medical teams must ensure patients have understood trial information, but this needs to go beyond biomedical information to give ongoing consideration to wellbeing and quality-of-life matters, including alternatives to anti-cancer treatment. Finally, as a society, we should also consider the vocabulary used surrounding cancer. Metaphors can be useful and are frequently used by patients. However, having undue expectations of patients to ‘fight’ cancer may not be a holistic or helpful approach. Allowing patients to define their own experience of disease can enable fulfilment without pressures to perform, aiding in a good death.

## Supplementary Information

**Additional file 1.**

**Additional file 2.**

## Data Availability

Not applicable. Included studies can be accessed using the citations provided.

## Abbreviations

ISRCTN Registry: International Standard Randomised Controlled Trial Number
ISSG: InterTASC Information Specialists’ Sub-Group
MeSH: Medical Subject Headings
P1: Phase 1
SoC: Sense of Coherence
