# Supplementary material for: Hope and meaning-making in phase 1 oncology trials: a systematic review and thematic synthesis of qualitative evidence on patient-participant experiences
Source: Trials. 2022 May 16;23:409. doi: 10.1186/s13063-022-06306-9 (PMC9112562; doi:10.1186/s13063-022-06306-9)
Supplement: Supplementary file 1 — Additional file 1. [file 13063_2022_6306_MOESM1_ESM.docx]

**Search Strategies**

Searches were run in December 2019 and rerun in February 2021

**Database: Ovid MEDLINE(R) ALL <1946 to December 02, 2019>**

**Search Strategy:**

**--------------------------------------------------------------------------------**

1 exp Clinical Trials, Phase I as Topic/ (5199)

2 phase 1 clinical trial*.tw. (813)

3 Clinical trial, phase 1.pt. (0)

4 ((investigation or experimental) adj (therap* or trial*)).tw. (6075)

5 phase 1 study*.tw. (1343)

6 ((early phase or phase 1 or phase one) adj (trial or study or research or analysis)).tw. (2218)

7 ((early phase or phase 1 or phase one) adj (investigation* or experiment*)).tw. (34)

8 or/1-7 (14072)

9 exp Neoplasms/ (3242801)

10 cancer.tw. (1607605)

11 (carcin* or neoplas* or tumour* or tumor* or growth* or malig* or leukaemia* or lymphoma* or leukemia* or adenocarcin* or lesion* or oncolog*).tw. (4285190)

12 or/9-11 (5701081)

13 8 and 12 (6473)

14 (view* or viewpoint* or attitude* or experience* or opinion* or expression* or thought* or perspective* or understand* or satisfaction* or qualitative* or perceive* or perception*).tw. (5169433)

15 13 and 14 (1754)

16 limit 15 to (english language and humans) (1438)

**Database: Ovid MEDLINE(R) <1946 to February Week 1 2021>**

**Search Strategy:**

**--------------------------------------------------------------------------------**

1 exp Clinical Trials, Phase I as Topic/ (5465)

2 phase 1 clinical trial*.tw. (769)

3 Clinical trial, phase 1.pt. (0)

4 ((investigation or experimental) adj (therap* or trial*)).tw. (5670)

5 phase 1 study*.tw. (1282)

6 ((early phase or phase 1 or phase one) adj (trial or study or research or analysis)).tw. (2107)

7 ((early phase or phase 1 or phase one) adj (investigation* or experiment*)).tw. (36)

8 or/1-7 (13765)

9 exp Neoplasms/ (3413352)

10 cancer.tw. (1491495)

11 (carcin* or neoplas* or tumour* or tumor* or growth* or malig* or leukaemia* or lymphoma* or leukemia* or adenocarcin* or lesion* or oncolog*).tw. (3983328)

12 or/9-11 (5381722)

13 8 and 12 (6441)

14 (view* or viewpoint* or attitude* or experience* or opinion* or expression* or thought* or perspective* or understand* or satisfaction* or qualitative* or perceive* or perception*).tw. (4770602)

15 13 and 14 (1736)

16 limit 15 to (english language and humans) (1572)

17 limit 16 to yr="2019 - 2021" (154)

**Database: EMBASE <1947-Present>**

**Search Strategy:**

**--------------------------------------------------------------------------------**

1 (phase 1 clinical trial* or phase one trial* or phase 1 study*).tw. (6013)

2 ((investigation or experimental) adj (therap* or trial*)).tw. (17813)

3 ((early phase or phase 1 or phase one) adj (trial or study or research or analysis)).tw. (6231)

4 ((early phase or phase 1 or phase one) adj (investigation* or experiment*)).tw. (45)

5 1 or 2 or 3 or 4 (25789)

6 malignant neoplasm/ (41483)

7 (cancer* or carcin* or neoplas* or tumour* or tumor* or growth* or malig* or leukaemia* or lymphoma* or leukemia* or adenocarcin* or oncolog* or lesion*).tw. (6828629)

8 6 or 7 (6833784)

9 (view* or viewpoint* or attitude* or experience* or opinion* or expression* or thought* or perspective* or understand* or satisfaction* or qualitative* or perceive* or perception*).tw. (6833711)

10 5 and 8 and 9 (3881)

11 limit 10 to (english language and humans) (3051)

12 conference.pt. (4402491)

13 11 not 12 (1650)

14 from 13 keep 1-1650 (1650)

**Database: Embase <1996 to 2021 February 15>**

**Search Strategy:**

**--------------------------------------------------------------------------------**

1 (phase 1 clinical trial* or phase one trial* or phase 1 study*).tw. (6767)

2 ((investigation or experimental) adj (therap* or trial*)).tw. (17196)

3 ((early phase or phase 1 or phase one) adj (trial or study or research or analysis)).tw. (7057)

4 ((early phase or phase 1 or phase one) adj (investigation* or experiment*)).tw. (52)

5 1 or 2 or 3 or 4 (26168)

6 malignant neoplasm/ (59230)

7 (cancer* orcarcin* or neoplas* or tumour* or tumor* or growth* or malig* or leukaemia* or lymphoma* or leukemia* or adenocarcin* or oncolog* or lesion*).tw. (4594842)

8 6 or 7 (4624901)

9 (view* or viewpoint* or attitude* or experience* or opinion* or expression* or thought* or perspective* or understand* or satisfaction* or qualitative* or perceive* or perception*).tw. (6523175)

10 5 and 8 and 9 (3643)

11 limit 10 to (english language and humans) (2909)

12 conference.pt. (4549504)

13 11 not 12 (1402)

14 limit 13 to yr="2019 - 2021" (189)

**Database: PsycINFO <1806 to November Week 4 2019>**

**Search Strategy:**

**--------------------------------------------------------------------------------**

1 ((investigation or experimental) adj (therap* or trial*)).tw. (1230)

2 phase 1 study*.tw. (45)

3 ((early phase or phase 1 or phase one) adj (trial or study or research or analysis)).tw. (108)

4 ((early phase or phase 1 or phase one) adj (investigation* or experiment*)).tw. (11)

5 1 or 2 or 3 or 4 (1348)

6 exp Neoplasms/ (50066)

7 cancer.tw. (59512)

8 (carcin* or neoplas* or tumour* or tumor* or growth* or malig* or leukaemia* or lymphoma* or leukemia* or adenocarcin* or lesion* or oncolog*).tw. (182446)

9 6 or 7 or 8 (227719)

10 (view* or viewpoint* or attitude* or experience* or opinion* or expression* or thought* or perspective* or understand* or satisfaction* or qualitative* or perceive* or perception*).tw. (2022475)

11 5 and 9 and 10 (67)

12 limit 11 to english language (65)

**Database: APA PsycInfo <2002 to February Week 2 2021>**

**Search Strategy:**

**--------------------------------------------------------------------------------**

1 (phase 1 clinical trial* or phase one trial* or phase 1 study*).tw. (84)

2 ((investigation or experimental) adj (therap* or trial*)).tw. (1008)

3 ((early phase or phase 1 or phase one) adj (trial or study or research or analysis)).tw. (113)

4 ((early phase or phase 1 or phase one) adj (investigation* or experiment*)).tw. (10)

5 1 or 2 or 3 or 4 (1159)

6 exp Neoplasms/ (44114)

7 (carcin* or neoplas* or tumour* or tumor* or growth* or malig* or leukaemia* or lymphoma* or leukemia* or adenocarcin* or oncolog* or lesion*).tw. (132973)

8 cancer.tw. (53142)

9 6 or 7 or 8 (171951)

10 (view* or viewpoint* or attitude* or experience* or opinion* or expression* or thought* or perspective* or understand* or satisfaction* or qualitative* or perceive* or perception*).tw. (1480716)

11 5 and 9 and 10 (68)

12 limit 11 to (english language and humans) [Limit not valid in APA PsycInfo; records were retained] (64)

13 limit 12 to yr="2019 - 2021" (3)

**
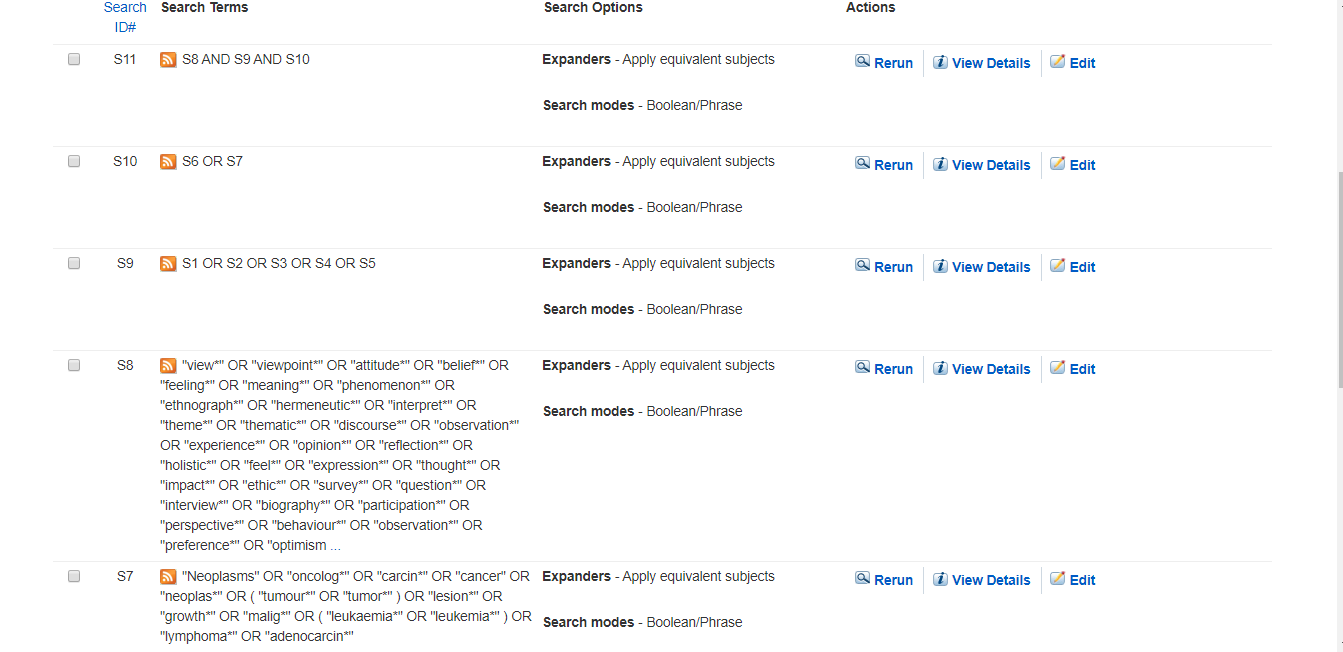

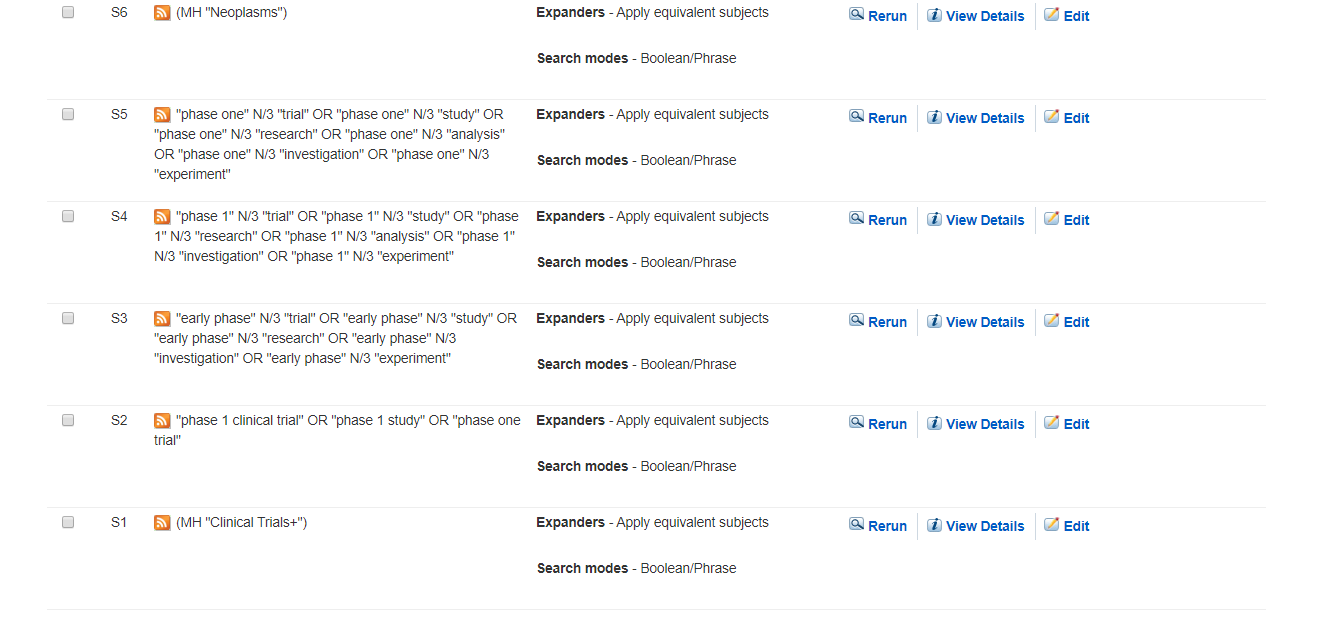
Ebsco CINAHL**

**Search Name: cochrane trails 10**

Date Run: 10/12/2019 11:46:13

Comment:

ID Search Hits

#1 MeSH descriptor: [Clinical Trials, Phase I as Topic] explode all trees 59

#2 (Neoplasms OR oncolog* OR carcin* OR cancer OR neoplas* OR tumour* OR tumor* OR lesion* OR growth* OR malig* OR leukaemia* OR leukemia* OR lymphoma* OR adenocarcin*):ti,ab,kw 279772

#3 (view* or viewpoint* or attitude* or experience* or opinion* or expression* or thought* or perspective* or understand* or satisfaction* or qualitative* or perceive* or perception*) 288026

#4 #1 AND #2 AND #3 10

**SCOPUS:**

( ( ( TITLE-ABS ( "phase 1 clinical trial" OR "phase one trial" OR "phase 1 study" ) ) OR ( TITLE-ABS ( "early phase" W/1 "trial" OR "study" OR "research" OR "analysis" OR "investigation" OR "experiment" ) ) OR ( TITLE-ABS ( "phase 1" W/1 "trial" OR "study" OR "research" OR "analysis" OR "investigation" OR "experiment" ) ) OR ( TITLE-ABS ( "phase one" W/1 "trial" OR "study" OR "research" OR "analysis" OR "investigation" OR "experiment" ) ) ) AND ( TITLE-ABS ( "Neoplasms" OR "cancer" OR "carcin*" OR "neoplas*" OR "tumour*" OR "tumor*" OR "growth*" OR "malig*" OR "leukaemia*" OR "lymphoma*" OR "leukemia*" OR "adenocarcin*" OR "lesion*" OR "oncolog*" ) ) ) AND ( ( TITLE-ABS ( "theme*" OR "thematic*" OR "discourse*" OR "observation*" OR "experience*" OR "opinion*" OR "reflection*" OR "holistic*" ) ) OR ( TITLE-ABS-KEY ( "view*" OR "viewpoint*" OR "focus group*" OR "attitude*" OR "belief*" OR "feeling*" OR "meaning*" OR "phenomenon*" OR "ethnograph*" OR "hermeneutic*" OR "interpret*" ) ) OR ( TITLE-ABS ( "feel*" OR "expression*" OR "thought*" OR "impact*" OR "ethic*" OR "survey*" OR "question*" OR "interview*" OR "biography*" ) ) OR ( TITLE-ABS ( "participation*" OR "perspective*" OR "behaviour*" OR "observation*" OR "preference*" OR "optimism*" OR "pessimism*" OR "understanding*" OR "communication*" OR "satisfaction*" ) ) OR ( TITLE-ABS ( "psychology*" OR "attitude*" OR "recount*" OR "understanding*" OR "narrative*" OR "qualitative*" OR "perceive*" OR "perception*" OR "questionnaire*" OR "focus group*" ) ) ) AND ( LIMIT-TO ( LANGUAGE , "English" ) ) AND ( LIMIT-TO ( DOCTYPE , "ar" ) ) AND ( LIMIT-TO ( SUBJAREA , "MEDI" ) ) 707
