# Supplementary material for: Hope and meaning-making in phase 1 oncology trials: a systematic review and thematic synthesis of qualitative evidence on patient-participant experiences
Source: Trials. 2022 May 16;23:409. doi: 10.1186/s13063-022-06306-9 (PMC9112562; doi:10.1186/s13063-022-06306-9)
Supplement: Supplementary file 2 — Additional file 2. [file 13063_2022_6306_MOESM2_ESM.docx]

Data Extraction Form-Phase 1 Review

| **General information** | | | |
| --- | --- | --- | --- |
|  | 1^st^ reviewer | | 2^nd^ reviewer |
| Data extracted by: |  | |  |
| Date: |  | |  |
| Data extraction checked by: |  | |  |
| Date: |  | |  |
| Citation: |  | |  |
| **Include:** | | **Exclude:** | |
| **Reason for exclusion:** | | | |
| **Study Information** | | |  |
| Study Aim: |  | |  |
| Study Setting:  *(location, country, context, where interview took place)* |  | |  |
| Study Design:  *(Qualitative, mixed methods)* |  | |  |
| Perspective:  *(those who are experiencing the situation – patients for this review)* |  | |  |
| **Population** |  | |  |
| Study Population:  *(no. of participants, age, gender, type of cancer, treatment agents)* |  | |  |
| Inclusion Criteria: | . | |  |
| Exclusion Criteria: |  | |  |
| **Methods** |  | |  |
| Data Collection:  *(Interviews, focus groups, survey free text data etc)* |  | |  |
| Role of the Researcher: |  | |  |
| Data Analysis:  *(Analysis method used)* |  | |  |
| **Findings** | | |  |
| Summary of study findings:  *(Include key themes)* |  | |  |
| Quotes from study: |  | |  |
| Conclusions: |  | |  |
| **Notes** |  | |  |
| Further Comments: |  | |  |
| **Further Information** | | | |
| Ethical approval: |  | |  |
| Funding sources: |  | |  |
| Conflicts of interest: |  | |  |
